# Non-Ablative Chemotherapy Followed by HLA-Mismatched Allogeneic CD3^+^ T-Cells Infusion Causes An Augment of T-Cells With Mild CRS: A Multi-Centers Single-Arm Prospective Study on Elderly Acute Myeloid Leukemia and int-2/High Risk Myelodysplastic Syndrome Patients

**DOI:** 10.3389/fonc.2021.741341

**Published:** 2021-10-13

**Authors:** Yan Huang, Minghua Hong, Zhigang Qu, Weiyan Zheng, Huixian Hu, Linjie Li, Ting Lu, Ying Xie, Shuangwei Ying, Yuanyuan Zhu, Lizhen Liu, Weijia Huang, Shan Fu, Jin Chen, Kangli Wu, Mingsuo Liu, Qiulian Luo, Yajun Wu, Fang He, Jingcheng Zhang, Junyu Zhang, Yu Chen, Minlei Zhao, Zhen Cai, He Huang, Jie Sun

**Affiliations:** ^1^ Zhejiang Province Engineering Laboratory for Stem Cell and Immunity Therapy; Zhejiang Laboratory for Systems & Precison Medicine, Zhejiang University Medical Center, Institute of Hematology, Bone Marrow Transplantation Center, The First Affiliated Hospital, Zhejiang University School of Medicine, Hangzhou, China; ^2^ Department of Hematology, Yiwu Central Hospital, Yiwu, China; ^3^ Department of Hematology, Affiliated Jinhua Hospital, Zhejiang University School of Medicine, Jinhua, China; ^4^ Department of Hematology, The Central Hospital of Lishui City, Lishui, China; ^5^ Department of Hematology, Taizhou Hospital of Zhejiang Province, Taizhou, China

**Keywords:** acute myelocytic leukemia, myelodysplastic syndrome, HLA-mismatched CD3^+^ T-cell infusion, cytokine release syndrome, graft-*versus*-host disease

## Abstract

**Objective:**

To evaluate the efficacy and safety of standard or low-dose chemotherapy followed by HLA-mismatched allogeneic T-cell infusion (allo-TLI) for the treatment of elderly patients with acute myeloid leukemia (AML) and patients with intermediate-2 to high-risk myelodysplastic syndrome (MDS).

**Methods:**

We carried out a prospective, multicenter, single-arm clinical trial. Totally of 25 patients were enrolled, including 17 AML patients and 8 MDS patients. Each patient received four courses of non-ablative chemotherapy, with HLA-mismatched donor CD3^+^ allo-TLI 24 h after each course. AML patients received chemotherapy with decitabine, idarubicin, and cytarabine, and MDS patients received decitabine, cytarabine, aclarubicin, and granulocyte colony-stimulating factor.

**Results:**

A total of 79 procedures were performed. The overall response rates of the AML and MDS patients were 94% and 75% and the 1-year overall survival rates were 88% (61–97%) and 60% (13–88%), respectively. The overall 60-day treatment-related mortality was 8%. Compared with a historical control cohort that received idarubicin plus cytarabine (3 + 7), the study group showed significantly better overall response (94% *vs.* 50%, *P*=0.002) and overall survival rates (the 1-year OS rate was 88% *vs.* 27%, *P*=0.014). Post-TLI cytokine-release syndrome (CRS) occurred after 79% of allo-TLI operations, and 96% of CRS reactions were grade 1.

**Conclusion:**

Elderly AML patients and intermediate-2 to high-risk MDS patients are usually insensitive to or cannot tolerate regular chemotherapies, and may not have the opportunity to undergo allogeneic stem cell transplantation. Our study showed that non-ablative chemotherapy followed by HLA-mismatched allo-TLI is safe and effective, and may thus be used as a first-line treatment for these patients.

**Clinical Trial Registration:**

https://www.chictr.org.cn/showproj.aspx?proj=20112.

## Introduction

Adoptive CD3^+^ T-cell infusion, such as donor lymphocyte infusion (DLI), is an established treatment strategy for activating donor-derived T-cells to eliminate leukemic cells (1, 2). The recent emergence of immune checkpoint inhibitors (3, 4) and chimeric antigen receptor T-cell immunotherapy technologies (5, 6) has led to a new understanding of the role of T-cell immunity in tumor treatment, including the abilities of genetically modified or unmodified T-cells with tumor antigen-recognition ability to kill tumor cells (7, 8). However, T-cell activation also triggers adverse reactions such as cytokine-release syndrome (CRS) (9, 10). Most gene-modified T-cell infusion (TLI) technologies are currently based on autologous T-cells, while most non-modified TLIs are based on allogeneic hematopoietic stem cell transplantation (allo-HSCT; i.e. infusion of donor-derived T-cells after allo-HSCT), and most of these infusions involve HLA-matched donor T-cells, also called donor lymphocyte infusion (DLI) (11, 12). Although some clinical trials of HLA-mismatched allogeneic TLI (allo-TLI) after non-myeloablative chemotherapy have shown encouraging effects (13–15), this approach is rarely used in clinical practice and the inherent immune mechanism of this technology remains unclear.

Elderly patients with acute myeloid leukemia (AML) respond poorly to current therapies and have low survival rates, with a remission rate of 43%–65% and a therapy-related mortality (TRM) rate of 11%–23% for patients older than 55 years (16–19), and a 5-year disease-free survival rate for elderly AML patients of only 15% (20). This poor prognosis for elderly AML patients is primarily related to more cytogenetic abnormalities and elderly individuals do not tolerate chemotherapy well (21). Myelodysplastic syndrome (MDS) is a hematological malignancy characterized by hematopoietic disorder, ineffective hematopoiesis, and an increased risk of progression to AML (22). Although MDS patients classified at intermediate-2 (int-2) to high risk of AML according to the International Prognostic Score System (IPSS) require chemotherapy (23), these patients are usually insensitive to or unable to tolerate chemotherapy. Long-term myelosuppression after chemotherapy is one of the treatment challenges in patients with MDS, leading to uncontrollable infection, bleeding, and an increased risk of TRM. Although HSCT has been considered as the only curative option for malignant hematologic disease, elderly AML patients or int-2 to high-risk MDS are often unable to receive HSCT due to advanced age, poor Eastern Cooperative Oncology Group (ECOG) scores, comorbidities, and a lack of suitable donors (24). There is thus an urgent need to develop appropriate treatments to maximize the response rate and reduce the incidence of adverse events in elderly patients with AML or MDS.

We designed a prospective, multicenter, single-arm clinical study to investigate a novel treatment for elderly AML patients and int-2/high-risk MDS patients. The protocol included four courses of non-ablative chemotherapy, each followed by one dose of HLA-mismatched allogeneic TLI (allo-TLI). We evaluated the response, survival, and safety, and observed the immune therapy-related reactions to explore the potential anti-leukemia mechanism of this novel T-cell therapy.

## Materials and Methods

### Patients and Donors

We enrolled 25 patients at three hospitals from October 27, 2017 to September 22, 2020. The patients were followed-up until March 2, 2021, with a median follow-up time of 30.0 months (range: 1.2-35.9 months). The inclusion criteria were as follows: 1) diagnosed with *de novo* AML and age ≥55 years, or *de novo* int-2 to high-risk MDS and age 18–80 years; 2) unfit (see **Supplementary Materials 1**) for or unwilling to undergo allo-HSCT; 3) ECOG performance status ≤2; and 4) available donor willing to donate T-cells. The diagnosis was defined according to the 2016 World Health Organization (WHO) criteria (25). The risk stratification of AML was assessed using the 2019 National Comprehensive Cancer Network (NCCN) guidelines, and the risk stratification of MDS was assessed using the IPSS system (23). Donor criteria were as follows: 1) ≤45 years old; 2) relative or legal relation of the patient (e.g. spouse); 3) consent to donate lymphocytes after fully informed; 4) match in fewer than seven of 10 HLA loci; 5) no blood-borne or bone marrow disease; and 6) tolerated the process of granulocyte colony-stimulating factor (G-CSF) mobilization and lymphocytes collection.

A historical control cohort comprised 30 elderly AML patients enrolled at the First Affiliated Hospital, Zhejiang University School of Medicine, from August 30, 2010, to December 26, 2016. Follow-up ended on August 21, 2021. The median follow-up time was 61.2 months (range: 0.2-62.2 months) and the median follow-up time for surviving patients was 61.2 months (range: 56.5-62.2 months). The inclusion criteria and risk stratification were the same as those for the AML patients in the current study. The diagnosis was defined according to the 2008 WHO criteria (26).

This study was approved by the central ethics review board (ERB) of the First Affiliated Hospital and each sub-ERB of Yiwu Central Hospital; Affiliated Jinhua Hospital, Zhejiang University School of Medicine and the Central Hospital of Lishui City, and was conducted according to the Declaration of Helsinki. All patients and donors provided written informed consent. The trial was registered prospectively in the China Clinical Trials Registry (http://www.chictr.org.cn) (ChiCTR-ONC-17011948) in July 2017.

### Treatment Design

**Figure 1:**
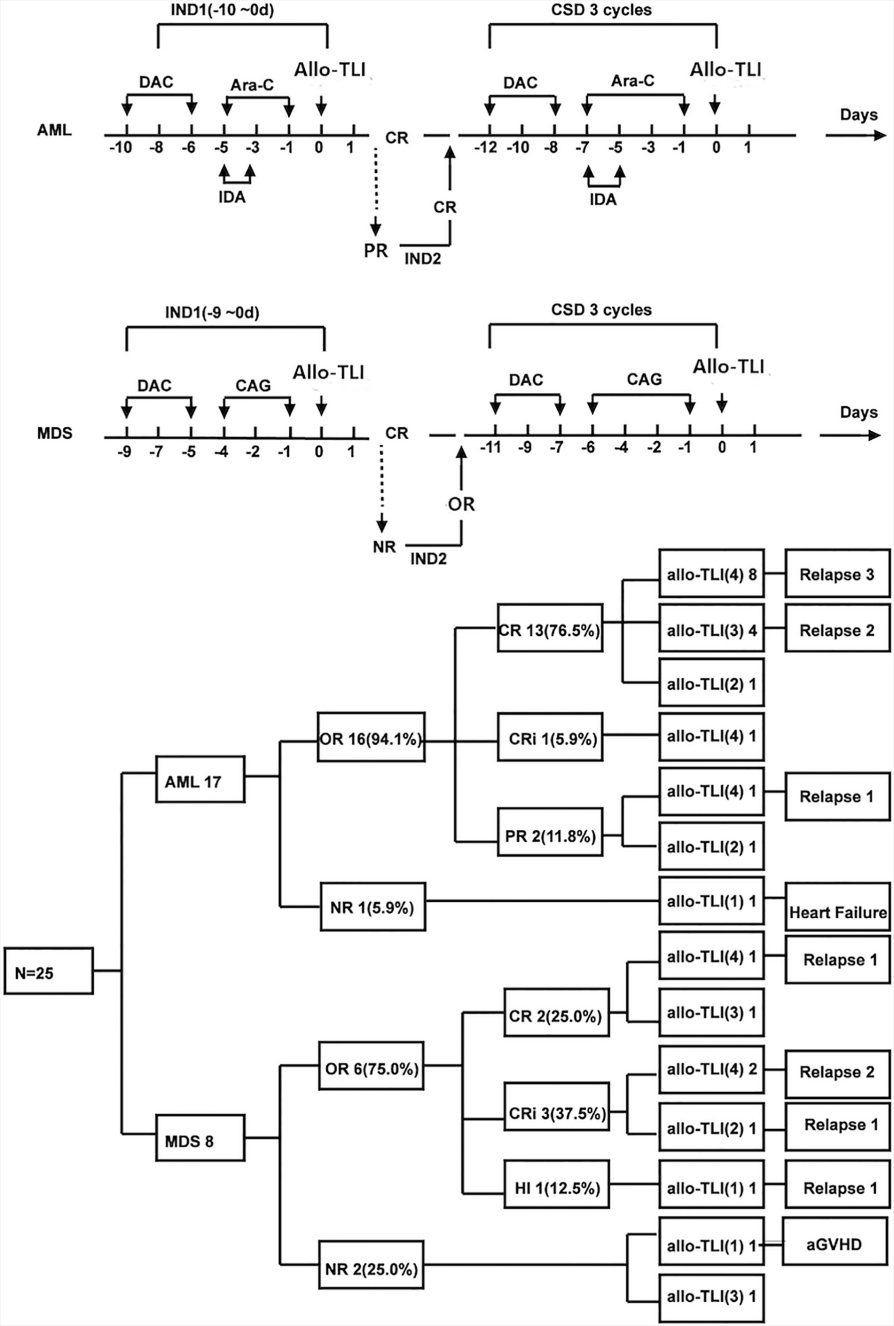
Protocol and results for elderly patients with *de novo* acute myeloid leukemia and patients with int-2/high-risk myelodysplastic syndrome treated with HLA-mismatched allogeneic CD3^+^ T-cell infusion. DAC, decitabine; Ara-C, cytarabine; IDA, idarubicin; CAG, cytarabine plus aclarubicin plus granulocyte colony-stimulating factor; CR, including complete remission and CR with incomplete blood count recovery; aGvHD, acute graft versus host disease; IND1, first induction treatment; CSD, consolidation treatment.

AML patients received induction therapy with decitabine (DAC, 15 mg/m^2^, day -10~-6) followed by a reduced dose of idarubicin 8 mg/m^2^, day -5~-3, and cytarabine 100 mg/m^2^, day -5~-1 (IA 3 + 5 regimen). Patients with MDS received DAC (15 mg/m^2^, day -9~-5) plus a 4-day induction chemotherapy regimen consisting of cytarabine (10 mg/m^2^, day -4~-1), aclarubicin (10 mg/day, day -4~-1), and G-CSF(150 μg every 12 h, day -5~-1). G-CSF was discontinued when the neutrophil count reached 2×10^9^/L. Patients who achieved partial remission (PR) received a second course of the same induction chemotherapy. AML patients who failed to achieve PR; or complete remission (CR) after the second course of induction chemotherapy will be withdrawn from the trial and received other treatments. For consolidation treatment, AML patients who achieved CR received three further courses of DAC+ (IA 3 + 7). MDS patients who achieved bone marrow or blood responses received three further DAC+ 6-day of CAG regimens. CD3^+^ T-cells from the same donor were allocated equally among four doses, with one dose administered per cycle without graft versus host disease (GvHD) prophylaxis 24 h (day 0) after the end of each chemotherapy. The interval between treatment cycles was 60 days. Patients who developed signs of CRS were given acetaminophen instead of steroids. Patients who relapsed or experienced severe adverse events were withdrawn from the trial. Patients who completed four allo-TLI cycles were followed up closely every 3 months. The allo-TLI protocol and patient outcomes are shown in **Figure 1**.

Patients in the historical control cohort received IA(3 + 7) (idarubicin 8 mg/m^2^ for 3 consecutive days and cytarabine 100 mg/m^2^ for 7 consecutive days). Hydroxyurea and hemapheresis were permitted as rescue treatments to control white blood cells. No allo-TLI was given after chemotherapy.

### Mobilizing and Collecting Donor CD3^+^ T-Cells

Donors received G-CSF 8 μg/kg/day for 5–6 consecutive days from day –4. Peripheral blood lymphocytes were harvested on day −1 and 0 by using a white blood cell apheresis procedure (COBE Spectra, Lakewood, Colorado, USA). After apheresis, the donor cells were divided equally into four aliquots; the first aliquot was used immediately and the remaining three were cryopreserved in liquid nitrogen. The median number of mononuclear cells was 2.58×10^8^/kg (IQR: 2.30–3.49×10^8^/kg) and the median number of CD3^+^ T-cells infused per course was 0.44×10^8^/kg (IQR: 0.36–0.60×10^8^/kg). The dose per Kg for the TLI refers to the weight of the patient.

### Micorchimerism Detection

Microchimerism (donor cells <1%) was initially examined using bone marrow sample of patients on day 7 of the first course of chemotherapy and before each of the next three courses of chemotherapy. Microchimerism was detected using real-time quantitative polymerase chain reaction (RQ-PCR) of the insertion-deletion polymorphism site (InDel) with a sensitivity of 0.001% (27, 28) (Dishuo Beacon, Shanghai, China). The detailed method of microchimerism detection is shown in **Supplementary Materials 2**. Donor chimerism was examined in short tandem repeats (STR) using semi-quantitative PCR (Dishuo Beacon, Shanghai, China) (29).

### Definition of Response, Endpoints, and Safety Evaluation Criteria

Treatment response in elderly AML patients was assessed according to the 2003 revised recommendations of the International Working Group (IWG) (30). MDS patients were assessed according to the 2006 IWG response criteria (31). Overall response (OR) was categorized as CR, CR with incomplete hematologic recovery (CRi), or PR for AML. Hematologic improvement (HI) was added for MDS patients. Overall survival (OS) and progression-free survival were used to evaluate survival. Toxicities were evaluated according to the National Cancer Institute Common Terminology Criteria for Adverse Events (NCI CTCAE), version 3.0. Glucksberg Clinical Stage and Grade of aGvHD (32) was used to evaluate the severity of aGvHD. The 60-day TRM rate was categorized as mortality without disease progression or death within 60 days after treatment. Non-recurrence mortality was defined as mortality not due to disease recurrence or progression. The hematopoietic recovery time was defined as the duration from the end of chemotherapy to an absolute neutrophil count ≥0.5×10^9^/L or platelet count was ≥20×10^9^/L for 3 consecutive days. CRS was graded according to the CRS revised grading system (33).

### Statistical Analysis

The web tool “StatBox” (https://www.cnstat.org/statbox) is used to estimate sample size (34). Statistical analysis and presentation were carried out using SPSS 24.0 (Statistical Product and Service Solutions, Armonk, New York, USA) and GraphPad Prism Version 7.00 (San Diego, California, USA). Survival curves were prepared using the Kaplan–Meier method. Longitudinal trends in temperature, T-cell numbers, and cytokine levels in peripheral blood from pre- to post-allo-TLI day 7 were assessed using mixed model repeated measures (Generalized Linear Mixed Model) (35) in patients who had completed three or four cycles of allo-TLI before November 1, 2019. Correlations between two groups of quantitative data that did not conform to a Gaussian distribution were analyzed by Spearman’s test. We compared the 30 patients in the historical cohort with 17 AML patients who received HLA-mismatched allo-TLI, and prepared survival curves using the Kaplan–Meier method. A two-tailed *P* value <0.05 was considered statistically significant.

## Result

### Clinical Characteristics of Patients and Donors

A total of 25 patients were included in this study, including 17 elderly AML patients and eight patients with IPSS int-2 or high-risk MDS. A total of 79 HLA-unmatched allo-TLI procedures were completed. Of the 25 patient/donor pairs, 16 (64%) were matched at fewer than five of 10 HLA alleles. Nine of the 17 AML patients (53%) were considered high-risk, 62% of the MDS patients were int-2 and 38% were high-risk. The detailed clinical characteristics are presented in **Table 1**. The historical cohort comprised 30 elderly AML patients (median age 66 years, IQR: 63–70 years; 16 males and 14 females). There was no significant difference in risk stratification between the current AML patients and controls. The detailed patient characteristics are listed in **Table 2**.

**Table 1:** Clinical features of patients and donors.

Variables	AML	MDS	Total
Patients	17	8	25
Patient age			
Median, IQR	66 (62-68)	65 (61-70)	65 (62-68)
Donor age			
Median, IQR	34 (26-38)	36 (30-42)	34 (28-40)
Patient sex			
Male	12	5	17
Female	5	3	8
Donor sex			
Male	12	7	19
Female	5	1	6
Matched-HLA loci^a^			
≥6/10	1	1	2
≤5/10	10	6	16
AML prognostic stratification^b^			
good	3		3
intermediate	5		5
poor	9		9
MDS prognostic stratification (IPSS)^b^			
intermediate-2		5	5
high-risk		3	3
Median number of stem cells infused (IQR)			
MNC, 10^8^/kg^c^	2.58 (2.34-3.25)	3.26 (2.06-3.57)	2.58 (2.30–3.49)
CD3^+^, 10^8^/kg^c^	0.44 (0.37-0.62)	0.41 (0.26-0.57)	0.44 (0.36–0.60)

AML, acute myeloid leukemia; MDS, myelodysplastic syndrome; HLA, human leukocyte antigen; IPSS, International Prognostic Score System; MNC, mononuclear cells; NK, natural killer cells.

a Peripheral blood samples from donors and recipients were collected and HLA-A, HLA-B, HLA-C, HLA-DRB1, and HLA-DQB1 alleles were genotyped by polymerase chain reaction using sequence-specific primers, before treatment.

b Prognostic risk groups defined by NCCN guidelines, version 2019.

c Recipient/patient weight.

**Table 2:** Patients characteristics; response, survival, and blood cell recovery times in patients with HLA-mismatched allogeneic T-cell infusion and historical controls.

Variables	IA (*n*=30), *n* (%)	Allo-TLI (*n*=17), *n* (%)	*P* value
Patient age			
Median, IQR	66 (63-70)	66 (62-68)	0.245
Patient sex			
Male/Female	16/14	12/5	0.247
Prognostic stratification^a^			0.673
good	3 (10)	3 (18)	
intermediate	10 (33)	5 (29)	
poor	17 (57)	9 (53)	
OR^b^	15 (50)	16 (94)	0.002
CR+CRi	14 (47)	14 (82)	0.017
CR	12 (40)	13 (77)	0.016
CRi	2 (7)	1 (6)	1.000
PR	1 (3)	2 (12)	0.606
1-year-OS rates (rate,95%CI)	27% (15-48%)	88% (61–97%)	0.014
Median days for peripheral blood cell recovery, days (95%CI)^c^			
ANC≥0.5×109/L	14.0 (11.8-16.1)	12.0 (11.4-14.3)	0.175
PLT≥20×109/L	12.7 (11.2-14.1)	10.0 (9.6-13.5)	0.066

IA, idarubicin and cytarabine; Allo-TLI, infusion of HLA-mismatched allogeneic T-cells; OR, overall response; CR, complete remission; CRi, CR with incomplete blood count recovery; PR, partial remission.

a Prognostic risk groups defined by the NCCN guidelines, version 2019.

b According to the 2003 revised recommendations of the International Working Group (IWG) for standardization of response criteria for AML. Overall response consisted of CR, CRi, and PR.

c Peripheral blood cell recovery time defined as the time from the end of the first course of chemotherapy to an absolute neutrophil count (ANC) ≥0.5×10^9^/L (or platelet count ≥20×10^9^/L) for 3 consecutive days.

### Response and Survival

**Figure 2:**
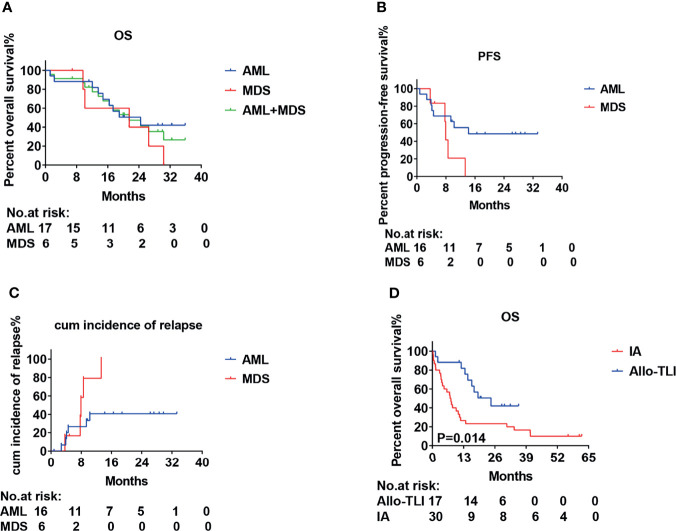
Survival and incidence of relapse curves for patients with acute myeloid leukemia or int-2/high-risk myelodysplastic syndrome treated with HLA-mismatched allogeneic CD3^+^ T-cell infusion. **(A)** Overall survival (OS); **(B)** Progression-free survival (PFS). **(C)** Cumulative incidence of relapse and **(D)** Overall survival (OS).

All patients were followed up until March 2, 2021. Thirteen (52%) patients (10 AML, 3 MDS) completed four courses of HLA-mismatched allo-TLI. For the induction chemotherapy, the CR+CRi rates in the AML and MDS groups were 82% and 63%, and the OR rates were 94% and 75%, respectively. The HI rate for MDS patients was 13% (**Table 3**). Two MDS patients failed to respond, including one with complicated karyotyping and a *TP53* mutation, and both chose to discontinue therapy. The 1-year OS rate and anticipated 2-year OS rate for the AML group were 88% (95%CI: 61%-97%) and 50% (95%CI: 25%-71%), and those for the MDS group were 60% (95%CI: 13%-88%) and 40% (95%CI: 5%-75%), respectively (**Figure 2A**). The median OS was 24.4 months for the AML group and was 21.5 months for the MDS group (**Table 3**). The 1-year progression-free survival (PFS) rates for the AML and MDS patients were 56% (95%CI: 29%-76%) and 21% (95%CI: 1%-60%) (**Figure 2B**), and the1-year cumulative incidence of relapse was 41% (95%CI: 13-67%) for the AML group and was 79% (95%CI: 49-93%) for the MDS group, respectively (**Figure 2C**). The overall non-recurrence mortality was 16% (4/25). One MDS patient died of grade 3 acute GvHD (aGvHD) after the first course of allo-TLI. One AML patient underwent HSCT after 13.4 months of continuous remission but died of aGvHD 3 months after HSCT. The incidence of aGVHD was 1/79 (1%). One AML patient died of heart failure during the first course of treatment. One AML patient died of acute pulmonary hemorrhage during the second course of treatment.

**Table 3:** Clinical outcomes of patients treated with microtransplantation.

Variables	AML	MDS
Patients	17	8
OR^a^ n (%)	16 (94)	6 (75)
CR n (%)	13 (77)	2 (25)
CRi n (%)	1 (6)	3 (38)
PR n (%)	2 (12)	0
HI n (%)	0	1 (13)
No response^b^ n (%)	1 (6)	2 (25)
Survival		
1-year OS % (95%CI)	88% (61–97%)	60% (13–88%)
2-year OS % (95%CI)	50% (25–71%)	40% (5-75%)
1-year PFS % (95%CI)	56% (29–76%)	21% (1–60%)
Median OS (months)	24.4	21.5
1-year cumulative incidence of relapse % (95%CI)^c^	41% (13-67%)	79% (49-93%)

AML, acute myeloid leukemia; MDS, myelodysplastic syndrome; OR, overall response; CR, complete remission; CRi, CR with incomplete blood count recovery; HI, hematological improvement.

a According to the 2003 revised recommendations of the International Working Group (IWG) for standardization of response criteria for AML or the 2006 IWG response criteria in myelodysplasia for MDS. Overall response consisted of CR, CRi, PR, and HI.

b Patients who did not reach OR were classified as no response.

c Relapse was defined as bone marrow blasts ≥5%, reappearance of blasts in peripheral blood, and/or extramedullary disease after CR or CRi.

This was a single-arm study, and we therefore used a historical cohort as a control group. The CR rates for the AML and historical groups were 77% *vs.* 40%, the CR+CRi rates were 82% *vs.* 47%, and the OR rates were 94% *vs.* 50%, respectively (*P*=0.016, 0.017 and 0.002, respectively). The 1-year OS rate was also significantly better compared with the historical controlgroup (88% *vs.* 27%; *P*=0.014) (**Table 2** and **Figure 2D**). The median OS was 7.6 months for the historical group and 24.4 months for the AML group.

### Adverse Events and Hematopoietic Recovery

All patients had grade 3–4 hematologic toxicity, including neutropenia and thrombocytopenia. Among non-hematologic toxicities, 32% of patients developed grade 3–4 infections. Severe adverse events involving major organs, such as respiratory failure, severe cardiac dysfunction, and renal dysfunction affected ≤2%. Severe liver dysfunction affected 4% of patients and was mainly attributed to antifungal drugs. The 60-day TRM rate of all patients was 8% (2/25), of AML patients was 6% (1/17). The median times to neutrophil and platelet recovery in the AML group were 12 days (95%CI: 11.4–14.3 days) and 10 days (95%CI: 9.6–13.5 days), and the equivalent times in the MDS group were 7 days (95%CI: 6.1–10.8 days) and 8 days (95%CI: 6.9–10.6 days), respectively. Details of the adverse events and hematopoietic recovery are shown in **Table 4**. There were no significant differences in the median times to neutrophil and platelet recovery between the current AML patients and the historical control group (all *P*>0.05) (**Table 2**).

**Table 4:** Adverse events^a^.

Variables	AML, *n (%)*		MDS, *n (%)*		Total, *n (%)*	
	≥grade 1 (%)	≥grade 3 (%)	≥grade 1 (%)	≥grade 3 (%)	≥grade 1 (%)	≥grade 3 (%)
Cases	57		22		79	
Hematological AE						
Neutropenia		57 (100)		22 (100)		79 (100)
Thrombocytopenia		57 (100)		22 (100)		79 (100)
Anaemia	8 (14)	40 (70)	2 (9)	17 (77)	10 (13)	57 (72)
Non-hematological AE						
Infection^b^	36 (63)	16 (28)	12 (55)	9 (41)	48 (61)	25 (32)
CRS	28/37 (76)	0	16/19 (84)	0	44/56 (79)	0
Dermatology/skin						
Rash	3 (5)	0	1 (5)	1 (5)	4 (5)	1 (1)
Pruritus/itching	1 (2)	0	0	0	1 (1)	0
Coagulation						
PTT prolongation	36 (63)	0	14 (64)	0	50 (63)	0
Fibrinogenopenia	21 (37)	0	4 (18)	0	25 (32)	0
Heart dysfunction	21 (37)	1 (2)	3 (14)	0	24 (30)	1 (1)
Liver dysfunction	23 (40)	2 (4)	6 (27)	1 (5)	29 (37)	3 (4)
Renal insufficiency	21 (37)	0	3 (14)	0	24 (30)	0
Acute GvHD^c^	0	0	1 (5)	1 (5)	1 (1)	1 (1)
Non- recurrent mortality	3 (18)		1 (13)		4 (16)	
60-days treatment related mortality	1 (6)		1 (13)		2 (8)	
Median days for peripheral blood cell recovery, days (95%CI)^d^						
ANC≥0.5×10^9^/L	12 (11.4-14.3)		7 (6.1-10.8)		11 (10.3-12.9)	
PLT≥20×10^9^/L	10 (9.6-13.5)		8 (6.9-10.6)		10 (9.3-12.3)	

AML, acute myeloid leukemia; MDS, myelodysplastic syndrome; AE, adverse event; PTT, partial thromboplastin time; CRS, cytokine-release syndrome; GvHD, graft versus host disease.

a According to National Cancer Institute Common Terminology Criteria for Adverse Events (NCI CTCAE), version 3.0.

b Infection (documented clinically) including intra-abdominal infection (1 patient), intestinal infection (1 patient), pneumonia (19 patients), and active tuberculosis (1 patient).

c According to the National Institutes of Health (NIH) consensus.

d Peripheral blood cell recovery time defined as time from the end of the first course of chemotherapy to an absolute neutrophil count (ANC) ≥0.5×10^9^/L (or platelet count ≥20×10^9^/L) for 3 consecutive days.

### Microchimerism

**Figure 3:**
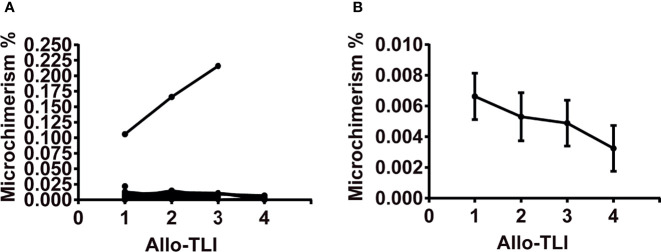
Changes in copies of microchimerism during four courses of HLA-mismatched allogeneic CD3^+^ T-cell infusion. **(A)** Changes in copies of microchimerism during four courses of HLA-mismatched allogeneic CD3^+^ T-cell infusion in 16 patients. **(B)** Copies of microchimerism decreased with increasing courses of HLA-mismatched allogeneic CD3^+^ T-cell infusion. Data represent mean and standard error. Microchimerism was examined on day 6 after the first course of HLA-mismatched allogeneic CD3^+^ T-cell infusion and before each of the following three infusions, using real-time polymerase chain reaction analysis of insertion-deletion polymorphism. Microchimerism was examined 41 times in 16 patients.

Nine patients were not willing to pay for microchimerism detection. A total of 16 patients were examined for microchimerism. Donor microchimerism was detected 37 out of 41 times (90%). Except for one MDS patient who had an average microchimerism of 0.163% (0.106–0.216%) of copies for all tests but no abnormal clinical symptoms or signs, all other patients displayed microchimerisms of 0.001%–0.022% of copies (**Figure 3A**). The average number of microchimerism copies decreased gradually with the increasing of allo-TLI courses (**Figure 3B**).

### GvHD

Only one patient (1/25, 4%) developed aGvHD. One patient developed grade 3 aGvHD on day 11 during the first course of HLA-mismatched allo-TLI. According to totally 79 HLA-mismatched allo-TLI procedures, the incidence of aGVHD was 1/79 (1%). The patient was a 65-year-old female diagnosed with MDS with 19% blasts, *TP53* mutation, and a complex karyotype. Her IPSS risk was int-2. She received a 5/10-matched HLA loci transplant and an infusion of 0.57×10^8^/kg CD3^+^ T-cells. This patient developed grade 3 aGvHD on day 11, with generalized erythematous pruritic rash, diarrhea (approximately 1.5–2 L) and liver dysfunction. A chimerism test on day 16 showed 52.07%. This patient failed to respond to anti-GvHD treatments and died of multiorgan failure on day 19. Compared with other patients, this patient had significantly increased levels of interferon (IFN)-γ and interleukin (IL)-17 after allo-TLI. The details of this patient are shown in **Supplementary Table 1**. No other patients developed acute or chronic GvHD.

### Post-HLA-Mismatched Allo-TLI CRS

**Figure 4:**
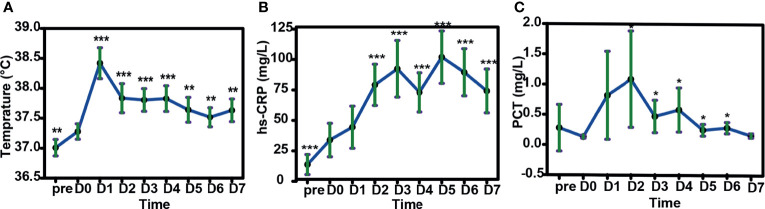
Changes in body temperature, peripheral blood high-sensitivity C-reactive protein (hs-CRP) and procalcitonin (PCT) in patients after HLA-mismatched allogeneic CD3^+^ T-cell infusion by mixed model repeated measures. **(A)** Changes in body temperature in patients after HLA-mismatched allogeneic CD3^+^ T-cell infusion. **(B)** Changes in hs-CRP and **(C)** PCT after HLA-mismatched allogeneic CD3^+^ T-cell infusion. **(D)** Changes in body temperature with increasing cycles of HLA-mismatched allogeneic CD3^+^ T-cell infusion. *P<0.05; **P<0.01; ***P<0.001 compared with day 0 **(A–C)**; compared with first cycle of HLA-mismatched allogeneic CD3^+^ T-cell infusion **(D)**. All data shown as estimated mean with 95% confidence intervals. pre, before chemotherapy; D0, day of donor T-cell infusion.

We examined the immune reactions after a total of 56 HLA-mismatched allo-TLI procedures. CRS was observed after 79% (44/56) of allo-TLI procedures, of which 96% (42/44) were grade 1 (**Table 4**). All patients with CRS had a body temperature >38°C, and this occurred within 24 h after allo-TLI in 39/56 (70%) of cases, with a temperature peak on day 1 (estimated mean: 38.4°C, 95%CI: 38.2–38.7°C; *P*<0.001 compared with day 0; **Figure 4A**). In addition to fever, high-sensitivity C-reactive protein (hs-CRP) levels were elevated to an average peak of 100.27 mg/L (95%CI: 78.78–121.76 mg/L; *P*<0.001 compared with day 0) on day 5 (IQR: day 2–6) (**Figure 4B**). Procalcitonin (PCT) was also increased to an average peak of 1.08 mg/L (95%CI: 0.28–1.88 mg/L) on day 2 (IQR: day 2–4) (**Figure 4C**). However, clinical manifestations, blood bacterial cultures, lung computed tomography, and other imaging examinations showed no infection during this period.

**Figure 5:**
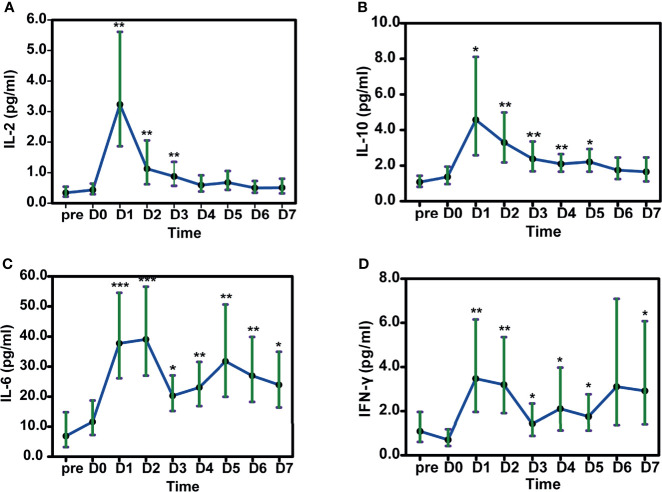
Changes in cytokines in patients after HLA-mismatched allogeneic CD3+ T-cell infusion analyzed by mixed model repeated measures. A IL-2. B IL-10. C IL-6. D IFN-γ. *P<0.05; **P<0.01; ***P<0.001 compared with D0. All data shown as estimated mean with 95% confidence intervals. pre: before chemotherapy; D0: day of donor T-cell infusion.

Levels of some cytokines were also elevated after HLA-mismatched allo-TLI. IL-2 increased with a peak on day 1 (estimated mean: 3.24 pg/mL, 95%CI: 1.86–5.61 pg/mL; *P*=0.002 compared with day 0) (IQR: day 1–3) (**Figure 5A**). IL-10 showed a similar trend, with a peak on day 1 (estimated mean: 4.57 pg/mL, 95%CI: 2.58–8.11 pg/mL; *P*=0.017; **Figure 5B**) (IQR: day 1–4). IL-6 peaked on day 2 (estimated mean: 39.08 pg/mL, 95%CI: 27.00–56.57 pg/mL; *P*<0.001) and remained high during the first week following allo-TLI (**Figure 5C**). IFN-γ levels also increased to their highest level on day 1 (estimated mean: 3.48 pg/mL, 95%CI: 1.96–6.15 pg/mL; *P*=0.005; **Figure 5D**) (IQR: day 1–5). However, levels of IL-4, IL-17A, and tumor necrosis factor (TNF)-α were not significantly changed (all *P*>0.05).

### Increase of Recipient-Derived Peripheral T-Cells After HLA-Mismatched Allo-TLI

**Figure 6:**
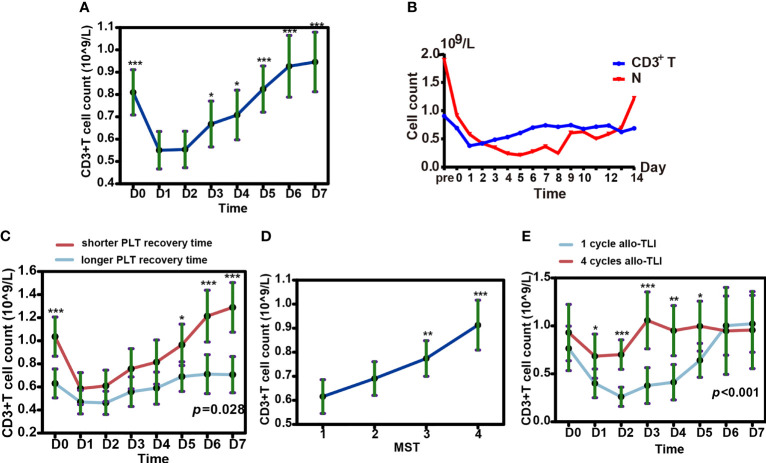
Changes in T cells and neutrophils in peripheral blood after HLA-mismatched allogeneic CD3^+^ T-cell infusion (allo-TLI). **(A)** Changes in CD3^+^ T-cells in peripheral blood from pre- to post-allo-TLI day 7. **(B)** Trends in median number of CD3^+^ T-cells and neutrophils (N) in peripheral blood after HLA-mismatched allo-TLI. **(C)** Changes in CD3^+^ T-cells in peripheral blood from pre- to post-allo-TLI day 7 in patients with different platelet (PLT) recovery times. longer recovery time (more than the median recovery time, ≥10 days for AML or ≥8 days for MDS); shorter recovery time (less than the median recovery time, <10 days for AML or <8 days for MDS). **(D)** Increase in CD3^+^ T-cells with more courses of allo-TLI. CD3^+^ T-cell counts were performed on D0 before each course of allo-TLI. **(E)** Changes in CD3^+^ T-cells in peripheral blood from pre- to post-allo-TLI day 7 in patients who completed one cycle of HLA-mismatched allo-TLI and in those who completed four cycles. Data represent the estimated mean with 95% confidence intervals. *P<0.05; **P<0.01; ***P<0.001 compared with D1 **(A)**; compared with one cycle of HLA-mismatched allo-TLI **(D)**; compared between two groups **(C, E)**.

Based on the 56 HLA-mismatched allo-TLI procedures, numbers of CD3^+^, CD4^+^, and CD8^+^ T-cells increased gradually to a peak on day 7 (*P*<0.001 compared with D0; **Figure 6A** and **Supplementary Figure 1**). Comparing the increasing curve of CD3^+^ T-cells with that for neutrophils showed that the increase in T-cells was not caused by hematologic recovery (**Figure 6B**). To determine the origin of the increased T-cells, we sorted peripheral blood T-cells from one patient. We analyzed the sorted T-cells by the chimerism detection, which confirmed that the increased peripheral T-cells were all derived from the recipient in this patient (see **Supplementary Table 3**). In addition, patients with shorter platelet recovery times (shorter than the median recovery time) had a higher number of CD3^+^ T-cells (*P*=0.028; **Figure 6C** and **Supplementary Table 4**). However, there was no significant difference in CD3^+^ T-cell counts between patients with shorter and longer neutrophil recovery times (*P*=0.967; **Supplementary Figure 2** and **Supplementary Table 4**). Additionally, patients who completed more cycles (3 or 4 cycles) of allo-TLI had significantly higher CD3^+^ T-cell counts than patients who completed only one cycle (*P*=0.001 and <0.001, respectively; **Figures 6D, E** and **Supplementary Table 4**).

## Discussion

DLI has been used as a type of allo-TLI for decades; however, non-ablative chemotherapy followed by HLA-mismatched stem cell transplantation, also referred to as “micro-transplantation”, has also been used to treat myeloid malignancies, with a better response, faster blood cell recovery, and lower TRM rate than conventional regimens, especially in elderly AML patients and patients with MDS (13–17, 19, 36–39). In these previous studies, although CD34^+^ hematopoietic stem cells were mobilized and isolated from HLA-mismatched donors and infused into the recipient, most researchers believed that the main functional anti-leukemia cells were T-cells, and donor CD3^+^ T-cells were found to interfere with survival after microtransplantation (38, 40). We therefore used HLA-mismatched allogeneic CD3^+^ TLI as immune therapy for elderly AML patients and int-2/high-risk MDS patients. Donor T-cell isolation was carried out according to the protocol for DLI in our institute (41, 42), resulting in a median infusion of 0.44×10^8^/kg CD3^+^ T-cells per dose, which was similar to the DLI dose regularly used in our institute for allogeneic stem cell transplantation (41). However, the optimal TLI dose for HLA-mismatched allo-TLI still needs to be determined. We used DAC plus IA(3 + 5) followed with HLA-mismatched allo-TLI for AML cohort and achieved a CR+CRi of 82%, a 1-year OS rate of 88%, and a median OS of 24.4 months. This result is significantly better than that of a historical control in our center, the OR rates were 94% *vs.* 50% for this study and the historical control, and the 1-year OS rate were 88% *vs.* 27%, respectively. We also used the DAC+CAG regimen for int-2/high-risk MDS patients, and showed an OR rate of 75% and a median OS of 21.5 months.

Most previous HLA-mismatched stem cell transplantation studies used standard-dose chemotherapy as induction therapy, followed by consolidation with high-dose cytarabine. In this study, we designed HMA plus reduced-dose IA for the AML cohort, which is based on the following considerations: Most of the elderly AML patients were secondary AML preceded by MDS or with myelodysplasia-related changes (43); poor-risk cytogenetics increased with advancing age, with 51% of patients older than 75 years reported to have poor-risk cytogenetics (44); In this study, 53% (9/17) of AML patients were in the unfavorable-risk category; According to the NCCN guidelines (version 3.2020), elderly AML patients with unfavorable-risk cytogenetics can be treated with a hypomethylating agent (HMA) or a standard dose of IA (3 + 7). We used DAC plus IA(3 + 5) followed with HLA-mismatched allo-TLI for AML cohort. *Zhou et al.* reported that DAC+IA(3 + 5) without allo-TLI had an induction CR rate of 64% in adult AML patients with myelodysplasia-related changes (45). The Bcl-2 inhibitor venetoclax plus HMA has also been recommended as first-line therapy for elderly AML patients with unfavorable-risk cytogenetics. Morsia et al. used venetoclax plus HMA in 44 newly diagnosed AML patients and achieved a 50% CR+CRi rate and median OS of 11 months (46). Our protocol also showed a significantly better response and survival compared to our historical controls. These results suggest that DAC+IA followed by HLA-mismatched allo-TLI improved response and survival rates in elderly AML patients, and can be an alternative therapy for “fit” elderly AML patients other than intensive chemotherapy or Bcl-2 inhibitor based therapies.

The CAG regimen, initially used by researchers in Japan, consists of low-dose cytarabine, low-dose aclarubicin, and G-CSF (47, 48), and has shown similar or better response/survival with lower treatment-related toxicity in AML and MDS patients (49–52). The DAC+CAG regimen has shown similar response and survival, but with reduced infections than standard regimens in elderly AML patients. A meta-analysis by Zhang et al. based on 10 randomized controlled trials including 590 int-2/high-risk MDS patients, found an OR rate of 70% (95% confidence interval: 58%–8l%) for DAC+CAG, which was significantly better than that for DAC or CAG alone (53). In the current study, the DAC+CAG regimen was used for int-2/high-risk MDS patients. Some new treatment strategies have recently demonstrated better responses than regular treatments in int-2/high-risk MDS patients. Ravandi et al. found that IA plus nivolumab achieved an 80% OR rate and 18.54-month median relapse-free survival in patients with newly diagnosed high-risk MDS (54). HSCT is first-line therapy for int-2/high-risk MDS if a suitable donor can be found. However, Kröger et al. found that, although HSCT achieved a high OR rate and long relapse-free survival, 32.3%–37.5% of cases developed grade 3–4 aGVHD (55). The current treatment protocol was thus similar to the above treatments, but with more excellent safety.

The current response and survival rates were similar to those reported in previous HLA-mismatched stem cell transplantation studies. Guo et al. used mitoxantrone plus cytarabine (MA 3 + 7) as induction chemotherapy and high-dose cytarabine as consolidation chemotherapy, resulting in CR induction of 80% and a 2-year OS rate of 39.3% (14). A multicenter study (13) of elderly patients with *de novo* AML used IA, MA, and DAC+CAG as induction and consolidation with high-dose cytarabine. The CR rates for 60–64- and 65–69-year-olds were 75.4% and 70.2%, and the equivalent 1-year OS rates were 87.7% and 85.8%, respectively. Hu et al. (15) used DAC plus mitoxantrone and cytarabine (MA)(3 + 7) as induction chemotherapy for patients with int-2 to high-risk MDS, followed with DAC plus high-dose cytarabine as consolidation chemotherapy, with an OR rate of 81% and 1-year OS rate of 94.1%. However, the median times to neutrophil and platelet recovery in their study were 14 and 17 days, respectively, which were longer than the 7 and 8 days in the current study. This suggests that standard or even low-dose, rather than high-dose, chemotherapies can be used as conditioning regimens for HLA-mismatched allo-TLI. However, a large cohort study with a randomized control group is needed to confirm our results.

Previous HLA-mismatched stem cell transplantation studies revealed that GvHD was rare, and the proportion of severe aGvHD was only 1.1% (13). GvHD prophylaxis is therefore not considered necessary. In the present study, one patient with MDS developed grade 3 aGvHD and eventually died. The incidence of aGVHD in this trial is similar as previously reported. There were no apparent differences between this patient and the other patients in terms of age (65 years), donor age (42 years), sex of donor/recipient (male/female), matched HLA loci (≤5/10), risk stratification of disease (MDS at IPSS int-2), and dose of infused CD3^+^ T-cells (0.57×10^8^/kg). GvHD used to be considered unavoidable in patients undergoing HLA-unmatched allo-HSCT, and GvHD prophylaxis was usually given even for HLA-matched allo-HSCT; however, some pioneer practices found that non-ablative conditioning followed by HLA-mismatched allo-HSCT produced donor–recipient chimerism, with no increase in GvHD, and long-term survival (56–58). The successful practice of microtransplantation has further expanded our understanding of HLA-mismatched allogeneic transplantation. The current study, demonstrated that, although aGvHD was rare, it can still occur after HLA-mismatched allo-TLI. Therefore close observation and early intervention are encouraged, and further studies are needed to improve our understanding of the mechanism of aGvHD.

This study showed that HLA-mismatched allo-TLI was generally safe, except for a very low risk of GvHD. The most common hematologic toxicities were neutropenia and thrombocytopenia. In previous studies of HLA-mismatched allo-HSCT, the median times to neutrophil and platelet recovery were significantly shorter than in the control group. The hematologic recovery time in the current study was similar to previous reports (14). However, there were no significant differences compared our current study with the historical control group. This might be because the historical control group did not include patients with DAC. Severe non-hematologic toxicities (> grade 3) were rare, and the 60-day TRM rates were 8% for all patients and 6% for AML patients. Mono-HMA has also been recommended for elderly AML patients unfit for intensive chemotherapy. A meta-analysis of nine published studies including 718 elderly patients with *de novo* AML showed that DAC monotherapy resulted in 30-day and 60-day TRM rates of 7% and 17%, respectively (59). Venetoclax plus HMA, as another popular treatment, was reported to have 30-day and 60-day TRM rates of 1% and 7%, respectively, compared with a 30-day TRM rate of 24% following intensive chemotherapy (60). These results indicated that the current treatment protocol was generally equivalent to non-intensive therapies in terms of safety.

The anti-leukemia mechanism of HLA-mismatched allo-TLI is not well known. Previous studies observed that the dose of infused donor CD3^+^ T-cells interfered with survival after HLA-mismatched allo-TLI. A CD3^+^ T-cell dose ≥1.1×10^8^/kg was associated with better OS and leukemia-free survival than a dose <1.1×10^8^/kg (38, 40). In this study, although we used G-CSF to mobilize donor stem cells, the highest CD3^+^ T-cell dose collected from a donor was 0.87×10^8^/kg (median 0.44×10^8^/kg). However, patients’ T-cell counts were significantly increased after HLA-mismatched allo-TLI, and this effect was not due to the recovery of bone marrow after chemotherapy. Based on the chimerism detection in one case, we suspected that the elevation in T-cells originated from the recipient. However, more cases should be enrolled to furtherly explore the origin of the increased T cells after allo-TLI. We also observed that the patient’s peripheral CD3^+^ T-cell counts increased in line with increasing cycles of HLA-mismatched allo-TLI, as reported by Zhu et al., suggesting that more cycles of allo-TLI promote graft versus leukemia effects (61).

Cytokine secretion is a byproduct of T-cell activation. Some cytokines are involved in the anti-tumor immunity of T-cells, while others contribute to the proliferation of hematopoietic cells. Previous studies showed changes in serum concentrations of several cytokines, such as IL-6, IL-8, and TNF-β, after HLA-mismatched allo-TLI (62). In the present study, IL-2, IL-10, IL-6, and IFN-γ levels increased significantly after HLA-mismatched allo-TLI, but IL-4, IL-17A, and TNF-α were unchanged. We also found that increased CD3^+^ T-cells levels were associated with platelet recovery time, and patients with shorter than median platelet recovery times had higher peripheral CD3^+^ T-cell counts. This might be due to an elevation of cytokines, which can promote the generation of platelets. IL-6 is considered to be involved in promoting megakaryocytic maturation and augmenting platelet counts (63), while other cytokines, such as IL-1 (64), IL-3, granulocyte-macrophage colony-stimulating factor (65), and IL-11 (66) stimulate platelet counts. However, whether these cytokines were released during post-allo-TLI CRS was unclear, and complete and detailed cytokine expression profiles need to be analyzed in future research.

In addition to activated T-cells and rising cytokine levels, we also observed a significant increase in body temperature after allo-TLI. Temperatures remained high during the first week after allo-TLI, and both hs-CRP and PCT increased during this period. Non-infectious fever, and increased hs-CRP, PCT, cytokines, and T-cells, are part of the immune response related to HLA-mismatched allo-TLI, similar to CRS following chimeric antigen receptor T-cell immunotherapy. Indeed, HLA-mismatched HSCT-related CRS was proposed as early as 2009 in a study of non-engraftment haploidentical cellular immunotherapy, termed “haplo immunostorm”, which presents with a series of clinical symptoms accompanied by cytokine flux, and has been shown to be sensitive to steroids (67). Sung et al. also reported an incidence of HLA-mismatched stem cell transplantation-related CRS of 40%, of which most cases were mild (62). There is currently no recognized term for this CRS, and we therefore referred to it as “post-TLI CRS”. In our study, 79% of cases developed post-TLI CRS, of which 96% were mild. Patients with CRS in this study received indomethacin rather than glucocorticoids in the first week after transfusion. Together, these findings further our understanding of the immune reactions following HLA-mismatched allo-TLI. However, a more formal post-TLI CRS assessment and grading system needs to be established.

This study still has many limitations. The cases number is small, especially for MDS patients. And this was not a randomized controlled study. There are many treatment options for elderly leukemia patients now. The controlled group could be regular intensive chemotherapy such as IA (3 + 7); intensive chemotherapy followed reduced intensity allogenic HSCT; BCL-2 based chemotherapies or HMA and low-dose cytarabine based chemotherapies, none of them was commonly accepted as “the best” first-line induction therapy for elderly AML patients, because of the high heterozygous property that the elderly AMLs have, the same do MDS patients. Although it has been reported that the incidence is very low, we had one case who developed aGvHD and died. The safety of this protocol still needs to be closely observed in future studies. This study has a pleased ORR, but the 1-year PFS rates for the AML and MDS patients were still low. A prospective clinical trial on a maintenance therapy followed four courses of allo-TLI has been registered, trying to overcome the relapse problem. More cases, well-designed randomized control studies should be working on in the following years.

In conclusion, the current study which enrolled 25 patients (17 AML and 8 MDS patients) showed standard or low-dose chemotherapy plus HLA-mismatched allo-TLI were associated with a higher treatment response rate, longer survival and favorable safety in elderly AML patient and int-2/high-risk MDS patients, compared with a historical control group in our center with IA (3 + 7), and with reported data on conventional treatments. Although this pilot study only enrolled 25 patients, we analyzed the results of 79 HLA-mismatched allo-TLI procedures. Based on these data, this study provides new insights into the mechanism of HLA-mismatched allo-TLI. Post-TLI CRS is common and usually mild, and a higher T-cell level after allo-TLI may be related to better blood cell recovery, while more cycles of HLA-mismatched allo-TLI may increase recipients’ T-cell levels, contributing to improved survival.

## Data Availability Statement

The original contributions presented in the study are included in the article/**Supplementary Materials**. Further inquiries can be directed to the corresponding authors.

## Ethics Statement

The studies involving human participants were reviewed and approved by Research Ethics Committee of the First Affiliated Hospital, College of Medicine, Zhejiang University. The patients/participants provided their written informed consent to participate in this study. Written informed consent was obtained from the individual(s) for the publication of any potentially identifiable images or data included in this article.

## Author Contributions

JS, HH, and ZC designed the study and revised the article. YH and MH performed the trials, processed the data analysis and interpretation and drafted the manuscript. ZQ, WZ, HXH, LJL, TL, YX, SY, YZ, LZL, WH, SF, JC, KW, ML, QL, YW, FH, JCZ, JYZ, YC, and MZ performed the trials and processed the data analysis. All authors contributed to the article and approved the submitted version.

## Funding

This work was supported by the National Natural Science Foundation of China (grant no. 82070200).

## Conflict of Interest

The authors declare that the research was conducted in the absence of any commercial or financial relationships that could be construed as a potential conflict of interest.

## Publisher’s Note

All claims expressed in this article are solely those of the authors and do not necessarily represent those of their affiliated organizations, or those of the publisher, the editors and the reviewers. Any product that may be evaluated in this article, or claim that may be made by its manufacturer, is not guaranteed or endorsed by the publisher.
