# Supplementary material for: Non-Ablative Chemotherapy Followed by HLA-Mismatched Allogeneic CD3+ T-Cells Infusion Causes An Augment of T-Cells With Mild CRS: A Multi-Centers Single-Arm Prospective Study on Elderly Acute Myeloid Leukemia and int-2/High Risk Myelodysplastic Syndrome Patients
Source: Front Oncol. 2021 Oct 13;11:741341. doi: 10.3389/fonc.2021.741341 (PMC8548743; doi:10.3389/fonc.2021.741341)
Supplement: Supplementary file 1 [file DataSheet_1.docx]

Supplementary Materials

# The criteria of unfit for intensive chemotherapy

Patients who were unfit for allogeneic stem cell transplantation should meet at least one of the following criterion: older than 70y; 2 to 3 status of Eastern Cooperative Oncology Group (ECOG) performance; ejection fraction ≤50% or congestive heart failure requiring treatment or chronic stable angina; reduced diffusion capacity of the lung with carbon monoxide ≤65%; creatinine clearance <45 mL/min; hepatic impairment with total bilirubin ＞1.5 × upper limit of normal; or any other comorbidity that was judged by physician to be incompatible with conventional intensive chemotherapy.

# Microchimerism detection

The microchimerism detection is based on real-time quantitative RT-PCR technology (RQ-PCR). We detect the InDel (Insertion or Deletion Polymorphisms) loci on 15 autosome chromosomes, they are N1-1, N1-2, N1-3, N2-1, N5-1, N5-4, N7-1, N9-1, N11-1, N11-2, N13-1, N13-2, N14-1, N16-2 and N21-1; and a sex chromosome site SRY.

# The sample size estimation

We used the web tool "StatBox" (https://www.cnstat.org/statbox) to estimate sample size (1), and SAS9.4 and PASS15 verify the reliability of the results. A significant difference test was used to estimate the number of cases to be enrolled in this study. For elderly *de novo* AML patients, the CR rate was approximately 35% for patients treated with DA protocol as induction (daunorubicin 45 mg/m^2^ for 3 consecutive days and cytarabine 200 mg/m^2^ for 7 consecutive days) (2). And in a multicenter analysis, the CR rate for those used allo-TLI plus IA, MA, or DAC+CAG as induction and consolidation with high-dose cytarabine was 75% (3). While for MDS patients with int-2/high risk, the CR rate for those treated with allo-TLI+ decitabine and cytarabine was 52.4% (4), and the CR rate for those treated with decitabine alone or combination with other chemotherapy agents was approximately 35% (5). Therefore, we hypothesized that the treatment strategy in our study could achieve a CR rate of 75% in *de novo* AML and 52% in MDS patients with int-2/high risk, which were simultaneously statistically different from that of treatment protocol without allo-TLI. The test level (α) was defined as 0.05 and the test efficiency (1-β) was defined as 0.80. Number of samples in historical control cohort: AML =2:1. According to Person Chi-square test, the sample sizes of AML patients and the historical control cohort to be included in this study were 15 and 30 (power = 0.8058). Furthermore, based on the variance of null hypothesis estimation and the standard approximation method, the sample sizes of MDS patients to be included in this study were 64 (power = 0.8026). The sample size of AML patients but not MDS patients included in this study met the estimated sample size, and 30 *de novo* AML patients treated with IA (3+7) protocol served as historical controls.

# Supplementary Figures and Tables

## Supplementary Figures

**
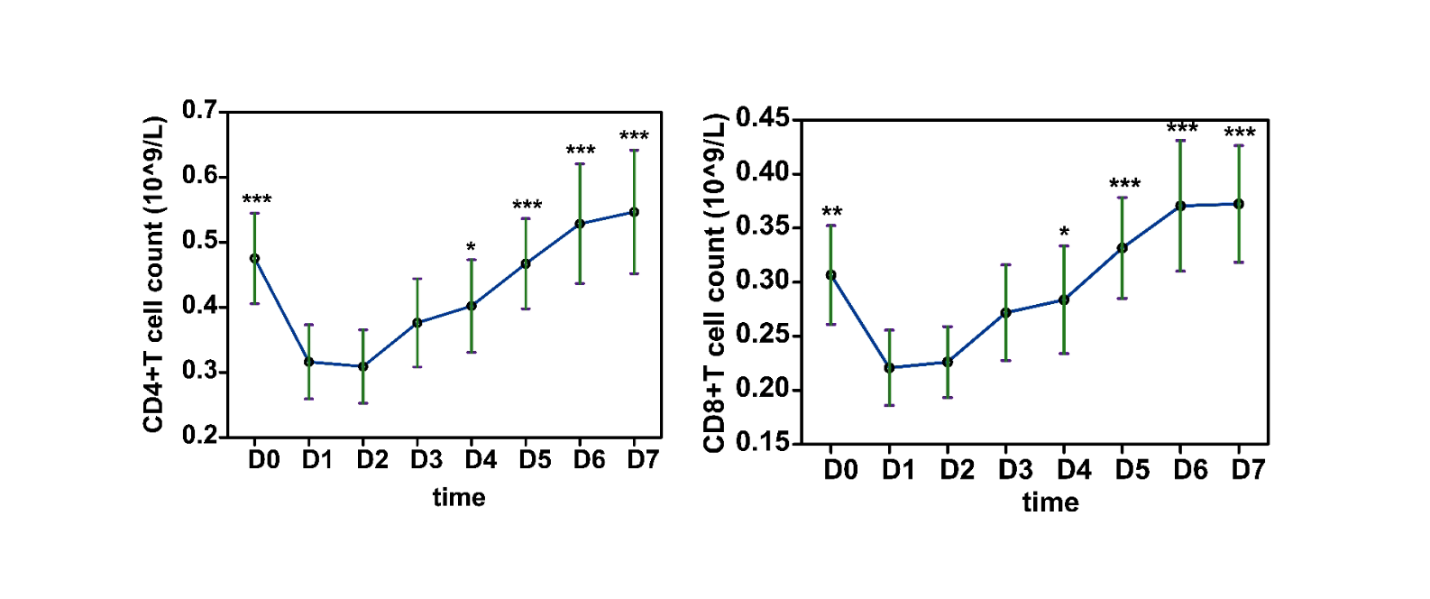
**

**Supplementary Figure 1.** Mean CD4^+^ and CD8^+^ T-cell numbers per unit volume in peripheral blood of patients from pre- to post-allo-TLI (day 7), analyzed by mixed model repeated measures. **P*<0.05; ***P*<0.01; ****P*<0.001 compared with D0. All data shown as estimated mean with 95% confidence intervals. D0: day of donor T-cell infusion.


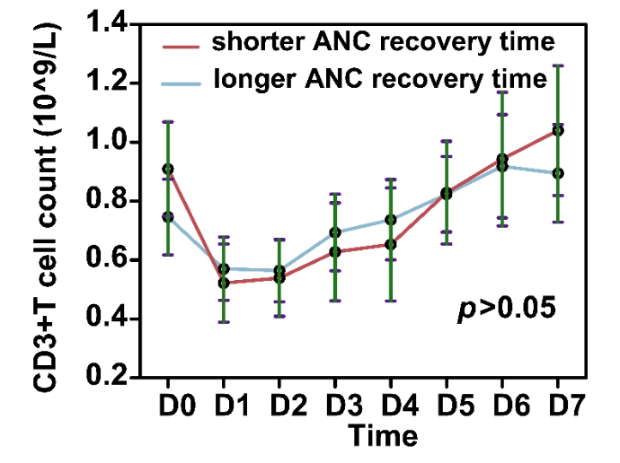


**Supplementary Figure 2.** Changes in CD3^+^ T cells in peripheral blood from pre- to post-allo-TLI (day 7) in patients with different absolute neutrophil (ANC) recovery times. Shorter ANC recovery time: less than the median ANC recovery time.

## Supplementary Tables

**Supplementary Table 1.** Clinical features of patient with graft versus host disease and other patients.

|  | **aGvHD Patient** | **Other MDS patients** | **All Other Patients** |
| --- | --- | --- | --- |
| Patients |  | 7 | 24 |
| Patient age |  |  |  |
| Median, IQR | 65 | 64(61-72) | 65(62-68) |
| Donor age |  |  |  |
| Median, IQR | 42 | 34(29-41) | 34(28-39) |
| Patient sex |  |  |  |
| Male |  | 5 | 17 |
| Female | Y | 2 | 8 |
| Donor sex |  |  |  |
| Male | Y | 6 | 19 |
| Female |  | 1 | 6 |
| MDS prognostic stratification (IPSS)^a^ |  |  |  |
| Intermediate-2 | 1 | 4 | 4 |
| High risk |  | 3 | 3 |
| Median number of stem cells infused (IQR) | |  |  |
| MNC, 10^8^/kg^b^ | 3.60 | 3.02(1.94-3.49) | 2.58(2.25-3.49) |
| CD3^+^, 10^8^/kg^b^ | 0.57 | 0.40(0.24-0.56) | 0.42(0.35-0.60) |

*aGvHD, acute graft versus host disease; AML, acute myeloid leukemia; MDS, myelodysplastic syndrome; HLA, human leukocyte antigen; IPSS, International Prognostic Score System; MNC, mononuclear cells; NK, natural killer cells.*

*^a^Prognostic risk groups defined by NCCN guidelines, version 2019.*

*^b^Recipient/patient weight.*

*This patient developed GvHD.*

**Supplementary Table 2.** The relationship between donor and recipient.

| Patients | | | Donors | | Relationship |
| --- | --- | --- | --- | --- | --- |
| Gender | Age | Diagnosis | Gender | Age |  |
| male | 71 | AML | male | 34 | son |
| female | 66 | AML | male | 21 | grandson |
| female | 64 | AML | male | 40 | son |
| male | 58 | AML | female | 33 | daughter |
| male | 55 | AML | male | 38 | son |
| male | 62 | AML | male | 19 | grandson |
| male | 63 | AML | male | 35 | son |
| male | 66 | AML | male | 39 | son |
| female | 71 | AML | male | 42 | son |
| male | 63 | AML | female | 31 | daughter |
| male | 62 | AML | male | 38 | son |
| male | 62 | MDS | male | 41 | nephew |
| female | 65 | MDS | male | 42 | son |
| female | 64 | MDS | male | 38 | son |
| male | 66 | MDS | male | 33 | son |
| male | 61 | MDS | female | 34 | daughter |
| male | 72 | MDS | male | 23 | grandson |
| male | 56 | MDS | male | 29 | son |
| male | 69 | AML | female | 23 | niece |
| female | 76 | AML | female | 20 | granddaughter |
| female | 73 | MDS | male | 42 | son |
| male | 67 | AML | male | 42 | son |
| male | 67 | AML | male | 27 | nephew |
| female | 67 | AML | male | 35 | Son |
| male | 59 | AML | female | 29 | daughter-in-law |

**Supplementary Table 3.** Chimerism detection of peripheral blood T cells in one patient.

|  | **Patient Pre-allo-TLI** | **Donor** | **Patient Post-allo-TLI^b^** |
| --- | --- | --- | --- |
| STR loci^a^ | | | |
| D8S1179 | 12 , 14 | 12 , 16 | 12 , 14 |
| D21S11 | 29 , 32.2 | 32.2 , 33.2 | 29 , 32.2 |
| D7S820 | 11 , 12 | 11 , 12 | 11 , 12 |
| CSF1PO | 10 , 12 | 10 , 12 | 10 , 12 |
| D3S1358 | 15 , 16 | 15 , 16 | 15 , 16 |
| D5S818 | 11 , 13 | 13 | 11 , 13 |
| D13S317 | 8 | 8 , 10 | 8 |
| D16S519 | 9 , 11 | 9 , 11 | 9 , 11 |
| D2S1338 | 19 , 24 | 24 | 19 , 24 |
| D19S433 | 14 , 14.2 | 14 , 14.2 | 14 , 14.2 |
| VMA | 14 , 17 | 16 , 17 | 14 , 17 |
| D12S391 | 15 , 19 | 19 , 20 | 15 , 19 |
| D18S51 | 14 , 15 | 15 | 14 , 15 |
| Amel | X , Y | X | X , Y |
| D6S1043 | 19 | 13 , 19 | 19 |
| FGA | 19 , 22 | 19 , 23 | 19 , 22 |

*STR, short tandem repeat; TLI, T-cell infusion.*

*^a^Donor chimerism was performed on STRs using semi-quantitative polymerase chain reaction.*

*^b^Day 6 after HLA-mismatched allo-TLI.*

**Supplementary Table 4.** Mixed model repeated measure analysis for CD3^+^T cells.

|  | **Mean of CD3^+^T cells(10^9^/L)** | **Sd** | ***P*** | **95%CI** | | |
| --- | --- | --- | --- | --- | --- | --- |
| Age | | | | | |  |
| ≥63y | 0.746 | 0.030 |  | 0.687 | 0.805 | |
| <63y | 0.849 | 0.031 | 0.011 | 0.787 | 0.910 | |
| Allo-TLI cycle | | | | | |  |
| 1 st cycle | 0.667 | 0.036 | x | 0.595 | 0.738 | |
| 2 nd cycle | 0.734 | 0.036 | 0.157 | 0.664 | 0.804 | |
| 3 rd cycle | 0.823 | 0.037 | 0.001 | 0.75 | 0.897 | |
| 4 th cycle | 0.965 | 0.052 | <0.001 | 0.863 | 1.066 | |
| ANC recovery time^a^ | | | | | |  |
| Longer recovery time | 0.798 | 0.028 |  | 0.744 | 0.852 | |
| Shorter recovery time | 0.796 | 0.035 | 0.967 | 0.728 | 0.865 | |
| PLT recovery time^b^ | | | | | |  |
| Longer recovery time | 0.664 | 0.029 |  | 0.607 | 0.722 | |
| Shorter recovery time | 0.930 | 0.034 | <0.001 | 0.862 | 0.998 | |

*^a^ANC recovery time: longer recovery time (more than the median recovery time, ≥13 days for AML or ≥7 days for MDS); shorter recovery time (less than the median recovery time, <13 days for AML or <7 days for MDS).*

*^b^PLT recovery time: longer recovery time (more than the median recovery time, ≥10 days for AML or ≥8 days for MDS); shorter recovery time (less than the median recovery time, <10 days for AML or <8 days for MDS).*

*TLI: T-cell infusion; ANC: absolute neutrophil count; PLT: platelet; AML, acute myeloid leukemia; MDS, myelodysplastic syndrome.*

*x: Other groups compared with this group.*

**Supplementary Table 5.** The follow-up time of the historical control group.

| Patients | Follow-up time（months） | Status |
| --- | --- | --- |
| 1 | 0.2 | Died |
| 2 | 0.4 | Died |
| 3 | 0.4 | Died |
| 4 | 0.7 | Died |
| 5 | 1.3 | Died |
| 6 | 1.4 | Died |
| 7 | 3.0 | Died |
| 8 | 3.5 | Died |
| 9 | 3.6 | Died |
| 10 | 3.9 | Died |
| 11 | 4.2 | Died |
| 12 | 4.9 | Died |
| 13 | 6.0 | Died |
| 14 | 7.2 | Died |
| 15 | 7.4 | Died |
| 16 | 7.7 | Died |
| 17 | 8.0 | Died |
| 18 | 8.4 | Died |
| 19 | 9.9 | Died |
| 20 | 10.8 | Died |
| 21 | 11.4 | Died |
| 22 | 11.8 | Died |
| 23 | 13.8 | Died |
| 24 | 31.0 | Died |
| 25 | 34.1 | Died |
| 26 | 40.7 | Died |
| 27 | 40.8 | Died |
| 28 | 56.5 | Alive |
| 29 | 61.2 | Alive |
| 30 | 62.2 | Alive |

Reference styles:

1. Chow S-C, Shao J, Wang H, Lokhnygina Y eds. *Sample Size Calculations in Clinical Research: Third Edition*. 3rd ed. Boca Raton: Chapman and Hall/CRC (2017). doi:10.1201/9781315183084

2. Löwenberg B, Ossenkoppele GJ, van Putten W, Schouten HC, Graux C, Ferrant A, Sonneveld P, Maertens J, Jongen-Lavrencic M, von Lilienfeld-Toal M, et al. High-dose daunorubicin in older patients with acute myeloid leukemia. *N Engl J Med* (2009) 361:1235–1248. doi:10.1056/NEJMoa0901409

3. Guo M, Chao NJ, Li J-Y, Rizzieri DA, Sun Q-Y, Mohrbacher A, Krakow EF, Sun W-J, Shen X-L, Zhan X-R, et al. HLA-Mismatched Microtransplant in Older Patients Newly Diagnosed With Acute Myeloid Leukemia: Results From the Microtransplantation Interest Group. *JAMA Oncol* (2018) 4:54–62. doi:10.1001/jamaoncol.2017.2656

4. Hu K-X, Sun Q-Y, Guo M, Qiao J-X, Yu C-L, Qiao J-H, Dong Z, Sun W-J, Zuo H-L, Huang Y-J, et al. A Study of Human Leukocyte Antigen Mismatched Cellular Therapy (Stem Cell Microtransplantation) in High-Risk Myelodysplastic Syndrome or Transformed Acute Myelogenous Leukemia. *Stem Cells Transl Med* (2016) 5:524–529. doi:10.5966/sctm.2015-0196

5. Kantarjian HM, O’Brien S, Shan J, Aribi A, Garcia-Manero G, Jabbour E, Ravandi F, Cortes J, Davisson J, Issa J-P. Update of the decitabine experience in higher risk myelodysplastic syndrome and analysis of prognostic factors associated with outcome. *Cancer* (2007) 109:265–273. doi:10.1002/cncr.22376
