# Supplementary material for: Non-Ablative Chemotherapy Followed by HLA-Mismatched Allogeneic CD3+ T-Cells Infusion Causes An Augment of T-Cells With Mild CRS: A Multi-Centers Single-Arm Prospective Study on Elderly Acute Myeloid Leukemia and int-2/High Risk Myelodysplastic Syndrome Patients
Source: Front Oncol. 2021 Oct 13;11:741341. doi: 10.3389/fonc.2021.741341 (PMC8548743; doi:10.3389/fonc.2021.741341)
Supplement: Supplementary file 2 [file DataSheet_2.docx]

Chemotherapy combined with non-ablative chemotherapy followed by HLA-mismatched allogeneic CD3^+^ T-cells infusion to treat elderly acute myeloid leukemia and int-2/high risk myelodysplastic syndrome patients

A multi-centers single-arm prospective study

Informed consent, the donor part

1. This is information on stem cell mobilization and collection of your T lymphocytes as adoptive immunotherapy for the patient. Please read carefully, ask any questions, and decide whether to agree to receive this operation. You have the right to know the properties and purpose, the risks involved, and the possible effects on the human body. Your doctor cannot perform this operation on you without your knowledge and written consent signed by you. You have the right to accept or reject this operation at any time.
2. This Lymphocytes mobilization operation includes the use of cell set stimulation factor (G-CSF) mobilization. There may be soreness, bone pain, low fever, headache, injection site reaction, and other side effects in the G-CSF mobilization process. In sporadic cases, spleen rupture will happen, due to individual differences. The mobilization process will be interrupted due to a severe reaction.
3. In general, the collection of 2-3 times up to collect a required number of lymphocytes, but individuals may need to collect more times.
4. An ultrasound-guided venipuncture may need to be carried out for patients with poor conditions of their peripheral venous patients.
5. The typical reactions during the lymphocytes collection process will be: the application of anticoagulant-related toxic side effects (fingers, lips or facial numbness; muscle spasms; convulsions; arrhythmia and chest tightness), local hematoma and inflammation after an intravenous puncture, fever, nausea, vomiting, bleeding, chest tightness, arrhythmia, electrolyte disorders. Rare risks include hemolysis, thrombosis, air embolism, secondary infections, cardiac arrest, and other unforeseen conditions. The mobilization process will be interrupted due to a severe reaction or machine failure.
6. In response to the situation mentioned above, we will take manages timely to minimalize the reactions，protect the patient and make sure the operation is safely carried on.
7. By signing, you agree that the medical students or internship doctors will observe during the inspection process. Your clinical data may be published without your identity for medical and educational purposes.
8. Please carefully read the informed consent form. To accurately understand the above information, your doctor will explain the whole procedure to you and answer all your questions.
9. Your signature below indicates that: (1) you have read, understood and agreed to the above; (2) your doctor has fully explained the above procedure to you, (3) you have fully understood all the information about the operation, and (4) you authorize and consent to receive the operation mentioned above.

| Participant's name | Participant's signature | Date |
| --- | --- | --- |
|  |  |  |
| Witness’ name | Witness’ signature | Date |
|  |  |  |
| Doctor’s name | Doctor’s signature | Date |
|  |  |  |
| Contact phone number of your doctor or designated person: |  | |
